# The Paranoia Question

**Published:** 1907-04

**Authors:** 


### THE PARANOIA QUESTION.

The absorbing interest taken in the Thaw trial prompts us to outline as briefly as possible, and necessarily in but a fragmentary manner, some of the aspects of the "paranoia question." There is probably no other problem of modern psychiatry so difficult to solve or even to present. The solution has been attempted by many alienists with varying degrees of satisfaction, but the point of view of to-day is far from that of a decade ago. It is instructive to realize that fifteen years ago the European institutions were filled with patients thought by able alienists to be suffering from paranoia, while to-day in many of the same institutions, perhaps even with the same directors, one cannot find a paranoiac without the aid of a magnifying glass. Whence this veering around, when human nature and its diseases have undergone little alteration for centuries at least, and the disorders of the mind of to-day are not much different from those of yesterday or even more than a century ago, when Vogel, in 1772, first introduced the term paranoia into psychiatry?

What one finds in the discussion of the paranoia problem, especially from English and American sources, is a lack of definition of what the various writers themselves mean by that term. The prevalent custom of making a word stand as a symbol for a disease process, while it may have some warrant in general clinical medicine, has relatively little justification in the study of disorders of the mind, where the varying and perpleoing symptom complexes tend to make our concepts of mental diseases very hazy. The fact of the matter is that practically every psychiatrist whose opinion is worth much tuses the term in a different sense, and any discussion of the problem should concern not the word paranoia, but the particular author's application of it. Were this method followed here, as it is more carefully observed on the Continent, much less confusion would be met with, and some advance made in the study of the diseased conditions themselves.

In former times delusional mental states, especially if of a persecutory nature, were termed paranoia without further specification, and in some instances the admissions of patients with "paranoia" have been as high as from seventy to eighty per cent. When it became realized, largely through the studies of Werner, Tanzi, Riva, and Wernicke, and of late particularly of Kraepelin, that delusions of persecution were frequent accompaniments if not prominent symptoms in a large number of mental disturbances, the diagnosis "paranoia" began to be made with diminishing frequency, and the delusions of persecution considered merely as a general symptom of defective reasoning power. Thus, such persecutory ideas were observed to be present in hysteria, in neurasthenia, in epilepsy, and in defectives; they followed recovery from the infectious diseases, measles, typhoid fever, influenza, and others; they were very common as a result of chronic intoxications, notably with alcohol, and often of lead, mercury, morphine, cocaine, and ergot poisoning. As a secondary effect of many acute psychoses, such as maniac depressive insanity, such delusions are prevalent, as well as in a number of the so-called adolescent insanities with intellectual deterioration, now so well grouped by Kraepelin and his followers as dementia præcox. In patients suffering from symptomatic depression, from involution melancholia, and very largely in senile deterioration, persecutory delusional states are known to occur. Thus, little by little, "paranoia" disappeared, and in the minds of a few—notably some extreme followers of Kraepelin—there are no patients presenting the so-called "paranoid" delusions who may not be classified in some one or other of the groups already outlined. The pendulum has swung to the other extreme.

When one attempts a construction, therefore, of our present views of the question, it is by no means a simple matter. The truth probably lies not at either extreme, and for practical purposes, both from the standpoint of psychiatry and from that of the law, one must recognize that the "paranoid" group as recognized by those who would take a moderate view may be conveniently divided into three general heads. As early as in 1883 Morselli described in clear and unmistakable terms a rudimentary, or abortive, paranoia, which has received the sanction of many if not most subsequent students. Such forms have been grouped by German as well as by French writers as those mild insanities in which the various "phobias," fixed ideas, and obsessions are the features, developing on a psychopathic foundation. Delusions of persecution are here very frequently encountered. Whether Friedmann's mild cases of paranoia with recovery recently described (Monatschrift fur Psychiatrie und Neurologie, No. 17, 1906) are to be here included is an open question. The group is a large one and much in need of further exact delineation. Certainly Bianchi's paraphrenia does not help with its purely accidental characteristics.

The old familiar forms of paranoia, the chronic delusional manias of fifteen years ago, are for the most part to be ranged with the second group—secondary paranoias. These are the postinfection and posttoxic forms now relegated to their proper position. Many of the acute "paranoid" states with good prognosis belong here, but since some develop into the chronic systematized forms with little intellectual defect, their consideration calls for rare judgment. The alcoholist, morphinist, and cocainist are also subject to secondary "paranoid" persecutory states which are grouped here as well. Probably the largest number of the secondary paranoias are to be relegated to the dementia "paranoid" group of Kraepelin, in which the intellectual deterioration, the disorder of attention, and the lowering of the emotional tone aid in making a more exact diagnosis of the condition. Inasmuch as the progress of this disease is often very slow, the diagnosis may present at times almost insuperable difficulties.

A third group includes the so-called primary paranoias. These originate as a rule upon a psycopathic basis, and are characterized largely by the development of delusional systems in the face of relative clearness. Acute and chronic forms are widely admitted. Whether the acute forms of Mendel and others are simple or hallucinatory, and the periodic forms of Ziehen are to be classed with Morselli's group or with the manic depressives, or are purely toxic secondary paranoias, is one of the fine questions in modern psychiatry. Inasmuch as a favorable prognosis is given in many of these, their forensic importance is self evident.

The chronic systematized forms, then, make up the remainder of this general group. As already outlined, they constitute for many authors the only paranoias with which they are acquainted. These are the chronic delusional insanities made classic in regicides, reformers, insane religious leaders, persecuted persecutors, and litigious and erotic paranoiacs. Inasmuch as the definition of this type includes the ideas of chronicity without early intellectual defect, the prognosis is foreshadowed in the very definition. It is admittedly bad, but judgment must always be suspended until the disease has been in progress for a number of years, and then the reports of recovery by numerous observers, notably the cases of Mendel, Meschede, Bleuler, Freyberg, and Bartels, of patients who have suffered from chronic systematized paranoia for a number of years, sometimes made as late as ten or fifteen years after admission, should lead even the most pessimistic of observers to admit the possibility of the curability of chronic systematized paranoia even in its severest types. Furthermore, the recent reports of Friedmann (l. c.) and of Gierlich (Archiv fur Psychiatrie, No. 40) make it imperative to reconstruct some of our opinions relative to the occurrence of mild cases of chronic systematized paranoia with a favorable outcome.—New York Medical Journal, March 9th, 1907

